# Antinociceptive and Anti-Inflammatory Activities of the Ethanol Extract of *Annona muricata* L. Leaves in Animal Models

**DOI:** 10.3390/ijms11052067

**Published:** 2010-05-06

**Authors:** Orlando Vieira de Sousa, Glauciemar Del-Vechio Vieira, José de Jesus R. G. de Pinho, Célia Hitomi Yamamoto, Maria Silvana Alves

**Affiliations:** 1 Departamento Farmacêutico, Faculdade de Farmácia e Bioquímica, Universidade Federal de Juiz de Fora, Campus Universitário, Martelos, 36036-330, Juiz de Fora, MG, Brazil; E-Mails: glauciemar@gmail.com (G.D.-V.V.); jose.pinho@ufjf.edu.br (J.J.R.G.P.); hytomani@yahoo.com (C.H.Y.); 2 Departamento de Análises Clínicas, Faculdade de Farmácia e Bioquímica, Universidade Federal de Juiz de Fora, Campus Universitário, Martelos, 36036-330, Juiz de Fora, MG, Brazil; E-Mail: alves_ms2005@yahoo.com.br

**Keywords:** *Annona muricata*, Annonaceae, antinociceptive activity, anti-inflammatory activity

## Abstract

Antinociceptive and anti-inflammatory activities of the ethanol extract from *Annona muricata* L. leaves were investigated in animal models. The extract delivered per oral route (p.o.) reduced the number of abdominal contortions by 14.42% (at a dose of 200 mg/kg) and 41.41% (400 mg/kg). Doses of 200 and 400 mg/kg (p.o) inhibited both phases of the time paw licking: first phase (23.67% and 45.02%) and the second phase (30.09% and 50.02%), respectively. The extract (p.o.) increased the reaction time on a hot plate at doses of 200 (30.77% and 37.04%) and 400 mg/kg (82.61% and 96.30%) after 60 and 90 minutes of treatment, respectively. The paw edema was reduced by the ethanol extract (p.o.) at doses of 200 (23.16% and 29.33%) and 400 mg/kg (29.50% and 37.33%) after 3 to 4 h of application of carrageenan, respectively. Doses of 200 and 400 mg/kg (p.o.), administered 4 h before the carrageenan injection, reduced the exudate volume (29.25 and 45.74%) and leukocyte migration (18.19 and 27.95%) significantly. These results suggest that *A. muricata* can be an active source of substances with antinociceptive and anti-inflammatory activities.

## 1. Introduction

*Annona muricata* L. (Annonaceae), commonly known as soursop, is found from Central America to South America, including the North, Northeast and Southeast regions of Brazil [1,2]. Traditionally, the leaves are used for headaches, insomnia, cystitis, liver problems, diabetes, hypertension and as an anti-inflammatory, antispasmodic and antidysenteric [1,2]. The decoction of the leaves have parasiticide, antirheumatic and antineuralgic effects when used internally, while the cooked leaves, applied topically, fight rheumatism and abscesses [1–3].

Among the chemical constituents found in *A. muricata,* the alkaloids (reticulin, coreximine, coclarine and anomurine) [4,5] and essential oils (β-caryophyllene, δ-cadinene, epi-α-cadinol and α-cadinol) [6,7] stand out. However, species of the Annonaceae family, including *A. muricata*, have also been targeted for investigation due to appurtenant substances in the acetogenins class [8] that have been isolated from different parts of the plant [9]. For example, annomuricins A and B, gigantetrocin A, annonacin-10-one, muricatetrocins A and B, annonacin, goniothalamicin [10], muricatocins A and B, annonacin A, (2,4-*trans*)-isoannonacin, (2,4-*cis*)-isoannonacin [11], annomuricin C, muricatocin C, gigantetronenin [12], annomutacin, (2,4-*trans*)-10R-annonacin-A-one, (2,4-*cis*)-10R-annonacin-A-one [13], annopentocins A, B and C, *cis*- and *trans*-annomuricin-D-ones [14], annomuricine, muricapentocin [15], muricoreacin and murihexocin C [16] and annocatacin A and B [17] were identified in the leaves. These acetogenins have cytotoxic properties against tumor cell lines [10–17] and molluscicidal activity [18]. In addition, *A. muricata* leaf extracts have antioxidant [19] and molluscicidal properties [20].

*A. muricata* ethnomedicinal use, especially for inflammation, rheumatism and neuralgy, still lacks scientifically supported pharmacological and clinical validation. In this sense, the aim of the present study was to investigate the antinociceptive and anti-inflammatory properties of the ethanol extract from *A. muricata* leaves using experimental animal models.

## 2. Results and Discussion

### 2.1. Acute Toxicity

At the doses administered per oral route (p.o.), the ethanol extract from *A. muricata* leaves was toxic to animals with LD_50_ of 1.67 g/kg (95% confidence intervals 1.24–2.26 g/kg). This result served as a parameter for dosage definition in the experiments of antinociceptive and anti-inflammatory activities.

### 2.2. Writhing Response Induced by Acetic Acid in Mice

Doses (p.o.) of 200 and 400 mg/kg of *A. muricata* extract significantly reduced (*p* < 0.01 and *p* < 0.001, respectively) the abdominal contortions induced by acetic acid to 57.87 ± 1.55 s and 39.62 ± 1.97 s compared to the respective control (67.62 ± 2.03 s) (Table 1).

### 2.3. Effects on Formalin-Induced Nociception in Mice

The intraplantar injection of formalin promoted a biphasic characteristic response (Table 2). The time spent licking in the first phase (0–5 min) was 86.62 ± 3.18 s and in the second phase (15–30 min) was 93.87 ± 2.73 s for the control group. After 60 min of treatment, doses (p.o.) of 200 and 400 mg/kg of extract significantly inhibited (*p* < 0.001) the first phase at 23.67 and 45.02% and the second phase at 30.09 and 50.20%, respectively, when compared to the control.

### 2.4. Effects on Hot-Plate Latency Assay in Mice

The *A. muricata* ethanol extract increased the latency time of mice exposed to the hot plate (Table 3). After 60 and 90 min of treatment, doses (p.o.) of 200 (30.77 and 37.04%) and 400 mg/kg (82.61 and 96.30%) increased significantly (*p* < 0.05 and *p* < 0.001, respectively) the latency time in the respective control group. Morphine proved to be a potent analgesic, increasing the latency time within the evaluation periods. Naloxone, an opioid antagonist, blocked the morphine action but did not completely alter the antinociceptive effect of the tested extracts.

### 2.5. Effects on Carrageenan-induced Edema in Rats

The *A. muricata* ethanol extract anti-inflammatory effect evaluated by the paw edema method induced by carrageenan is shown in Table 4. Edema inhibition was observed 3 h after carrageenan application of doses (p.o.) of 200 (0.73 ± 0.06; 23.16 %; *p* < 0.05) and 400 mg/kg (0.67 ± 0.04; 29.47 %; p < 0.01). 4 h after carrageenan injections, the doses of 200 (0.53 ± 0.03; *p* < 0.01) and 400 mg/kg (0.47 ± 0.02; *p* < 0.001) reduced the respective paw edema (29.33 and 37.33%). In this time, indomethacin also reduced the paw edema (42.67%).

### 2.6. Effects on Carrageenan-Induced Pleurisy in Rats

**Figure 1.**
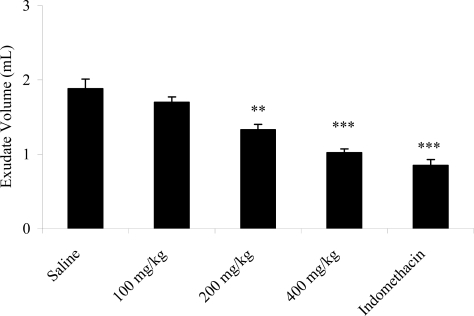
Effects of the ethanol extract from *A. muricata* leaves on pleural exudation induced by carrageenan in rats. Data are mean ± s.e.m. of six rats. **P < 0.01, ***P < 0.001 *vs.* control group.

**Figure 2.**
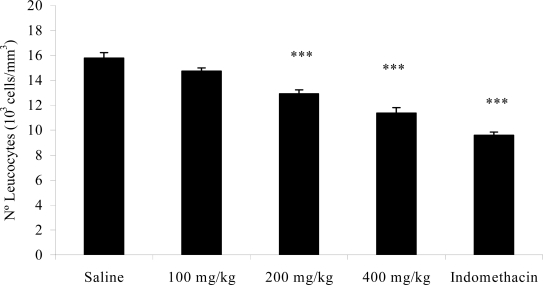
Effects of the ethanol extract from *A. muricata* leaves on number of leucocytes in carrageenan-induced pleurisy in rats. Data are mean ± s.e.m. of six rats. ***P < 0.001 *vs.* control group.

The pleurisy effects demonstrated that doses (p.o.) of 200 (p < 0.01) and 400 mg/kg (p < 0.001) of the extracts significantly reduced the exudate volume (Figure 1) and the number of total leukocytes (Figure 2). The exudate volume was decreased by 29.25 and 45.74% at doses (p.o.) of 200 and 400 mg/kg compared to the respective control. Leukocyte migration inhibition also occurred from doses (p.o.) of 200 (12.91 ± 0.32 × 10^3^ cells/mm^3^; p < 0.001) and 400 mg/kg (11.37 ± 0.44 × 10^3^ cells/mm^3^; p < 0.001). Indomethacin reduced the exudate volume and the leukocyte migration.

The acute toxicity test showed that the *A. muricata* leaves ethanol extract doses tested were toxic to mice. However, the largest dose administered (400 mg/kg) is less than the lowest dose applied for determination of the LD_50_ (0.5 g/kg or 500 mg/kg). Studies have demonstrated that isolated acetogenins from the *A. muricata* leaves are toxic to tumor cells [11–17] and molluscicides [18]. It is possible that the toxic effect of the ethanol extract could be due to the presence of these substances. However, the pharmacological doses definition of the ethanol extract was not described in the literature. In the present study, the LD_50_ was used to define the doses that were administered to the animals.

Based on the pharmacological tests results, the *A. muricata* ethanol extract has antinociceptive and anti-inflammatory activities, being firstly reported in the literature. Intraperitoneal administration of acetic acid releases prostaglandins and sympathomimetic system mediators like PGE_2_ and PGF_2α_ and their levels were increased in the peritoneal fluid of the acetic acid induced mice [21]. Thus, the antinociceptive effect of the ethanol extract could be mediated by peripherical effects, including the prostaglandin synthesis inhibition. The antinociceptive effect was also demonstrated by the biphasic response time of paw licking induced by formalin [22]. The first phase (0 to 5 min) corresponds to the neurogenic stage as an intensely painful process for the activation of nociceptive pathways, while inflammation mediators are produced after 15 minutes of formalin application (second phase) [22,23]. Substance P and bradykinin act as mediators in the first phase, while histamine, serotonin, prostaglandin and bradykinin are involved in the nociceptive response of the second stage [23]. The central action was confirmed in the hot plate test (200 and 400 mg/kg), showing that the maximum effect is reached after 90 minutes. This test is considered to be sensitive to drugs acting at the supraspinal modulation level of the pain response [24], suggesting at least a modulatory effect of the extract. In this study, antinociceptive action did not depend entirely on the opioid system, because naloxone treatment did not completely reverse the produced effect [25,26]. The formalin induced algesia test also indicated a possible anti-inflammatory activity (the second phase was reduced from 200 mg/kg).

The anti-inflammatory activity was confirmed by the paw edema induced by carrageenan in rats, a model widely used to study anti-inflammatory substances. Carrageenan induces paw edema resulting in the release of mediators such as histamine, serotonin, bradykinin, substance P and a platelet activating factor and prostaglandins [27–33]. In this study, oral treatment with the *A. muricata* extract significantly inhibited the paw edema. This evidence suggests that the anti-inflammatory actions of the ethanol extract are related to inhibition of one or more signaling intracellular pathways involved with these mediators effects.

Pleurisy produced by intrapleural injection of carrageenan leads to the formation of exudate in the pleural cavity [34,35] and leukocyte migration [35,36]. It is a method that assesses the inflammatory infiltrate and confirms the obtained paw edema results. Non-steroidal anti-inflammatory drugs, such as indomethacin, inhibit the accumulation of exudates and mobilization of leukocytes between 3 and 6 h after application of carrageenan [35,37]. By reducing the volume of exudate and the leukocyte migration, the *A. muricata* ethanol extract confirmed the results of the paw edema (Table 4 and Figures 1 and 2).

Plants belonging to the Annonaceae family have been investigated for its antinociceptive and anti-inflammatory properties [25,26,38]. However, considering the compounds isolated from *A. muricata*, these properties are not been reported for the alkaloids [4,5] and acetogenins [8–18]. Antinociceptive and anti-inflammatory activities have been attributed to essential oil of *Dennettia tripetala* (Annonaceae) [38], but such activities are not described for the major components [6,7] identified in *A. muricata*. Additional studies are necessary to establish the possible correlation between activities and chemical composition of this plant.

## 3. Experimental Section

### 3.1. Plant Material and Extraction

The plant material used in this study was collected in Juiz de Fora, State of Minas Gerais, Brazil, in February 2008. The species was identified by Dr Fátima Regina Gonçalves Salimena and a voucher specimen (CESJ number 48236) was deposited in the Herbarium of the Universidade Federal de Juiz de Fora, Brazil. Dried and powdered leaves (600 g) were exhaustively extracted in 95% ethanol (2.5 L) by static maceration for 3 weeks at room temperature with renewal of solvent every 2 days. The ethanol extract was filtered and evaporated under a rotary evaporator at controlled temperature (50–60 °C). This material was placed in a desiccator with silica to yield 36.40 g. The dried extract was dissolved using 1% DMSO in normal saline for pharmacological studies.

### 3.2. Chemicals

Drugs and reagents used in this study (and their sources) were as follows: acetic acid (Vetec Química Farm Ltda, Rio de Janeiro, RJ, Brazil), formaldehyde (Reagen Quimibrás Ind. Química S.A., Rio de Janeiro, RJ, Brazil), morphine hydrochloride (Merck Inc., Whitehouse Station, NJ, USA), naloxone and indomethacin (Sigma Chemical Co, St Louis, MI, USA).

### 3.3. Animals

Male Wistar rats (90–110 days) weighing 200–240 g and male Swiss albino mice (50–70 days) weighing 25–30 g were used in the experiments. The animals were provided by the Central Biotery of the Universidade Federal de Juiz de Fora. The animals were divided into groups and kept in plastic cages (47 × 34 × 18 cm) under a 12 h light/12 h dark cycle at room temperature (22 ± 2 °C), with free access to Purina rations and water. Animal care and the experimental protocol followed the principles and guidelines suggested by the Brazilian College of Animal Experimentation (COBEA) and were approved by the local ethical committee.

### 3.4. Acute Toxicity

Groups of ten mice received oral doses of 0.5, 1, 1.5, 2 and 3 g/kg of ethanol extract from *A. muricata*, while the control group received the vehicle (saline). The groups were observed for 48 h and mortality at end of this period was recorded for each group [39]. The LD_50_ (50% lethal dose) was determined by probit test using a log plot of percentage death *versus* dose [40]. The determination of LD_50_ served to define the doses used in experiments of pharmacological activities.

### 3.5. Acetic Acid-Induced Writhing Response in Mice

Antinociceptive activity was evaluated using the test of abdominal writhing induced by acetic acid in mice [41]. Animals were divided into groups of eight mice. Control mice received an i.p. injection of acetic acid 0.6% (0.25 mL) and 10 min later the writhes were counted over a period of 20 min. One group of mice received indomethacin (10 mg/kg) by the per oral route (p.o.) as a reference compound, and the other three groups received the extract at doses (p.o.) of 100, 200 and 400 mg/kg, 1 h before the acetic acid injection.

### 3.6. Formalin-Induced Nociception in Mice

Mice received subplantar injections of 20 μL 2.5% formalin (in 0.9% saline) and the time of paw licking (in seconds) was determined over 0–5 min (first phase - neurogenic) and 15–30 min (second phase - inflammatory) after formalin injection [22]. Animals (n = 8) were pretreated p.o. with extract (100, 200 or 400 mg/kg; 0.1 mL per 10 g body weight) or the reference compound, subcutaneous morphine (1 mg/kg), 1 h before administration of formalin. Control animals were treated with sterile saline (10 mL/kg).

### 3.7. Hot-Plate Latency Assay in Mice

Animals were placed on a hot-plate (Model LE 7406, Letica Scientific Instruments, Barcelona, Spain) heated at 55 ± 1 °C [42]. Three groups of mice (n = 8) were treated p.o. with ethanol extract (100, 200 or 400 mg/kg; 0.1 mL per 10 g body weight); the control group received sterile saline (10 mL/kg). Measurements were performed at time 0, 30, 60 and 90 min after drug administration, with a cut-off time of 40 s to avoid lesions to the animals’ paws. The effect of pretreatment with naloxone (1 mg/kg, subcutaneously) on the analgesia produced by the ethanol extract (400 mg/kg) was determined in a separate group of animals. Morphine (1 mg/kg, subcutaneously), in the absence and presence of naloxone treatment, was used as a reference.

### 3.8. Carrageenan-Induced Edema in Rats

Anti-inflammatory activity was assessed on the basis of inhibition of paw edema induced by the injection of 0.1 mL of 2% carrageenan (an edematogenic agent) into the subplantar region of the right hind paw of the rat [43]. Male Wistar rats were divided into groups of six animals which received p.o. doses of extract (100, 200 and 400 mg/kg; 0.1 mL per 10 g body weight), saline or indomethacin (10 mg/kg) 1 h before the injection of carrageenan. In the left paw, used as a control, 0.1 mL of sterile saline was injected. 1, 2, 3 and 4 h after injection of carrageenan, the measure of edema was made by the difference between the volume displaced by the right paw and the left paw using a plethysmometer (model LE 7500, Letica Scientific Instruments, Barcelona, Spain).

### 3.9. Carrageenan-Induced Pleurisy in Rats

Pleurisy was induced in male Wistar rats by intrapleural administration of 0.5 mL 2% carrageenan suspension in saline solution between the third and fifth ribs on the right side of the mediastinum [37]. Extract (100, 200 and 400 mg/kg), saline or indomethacin (10 mg/kg) p.o. were given 60 min before injection of the irritant. Animals were killed 4 h after carrageenan injection, and the skin and pectoral muscles were retracted. A longitudinal incision was made between the third and fifth ribs on each side of the mediastinum. The exudate was collected and transferred to a 15 mL conical centrifuge tube and the total volume determined. A 50 μL aliquot of the exudate was used to determine the total leucocyte count in Neubauer chambers.

### 3.10. Calculations and Statistical Analysis

Data are expressed as mean ± s.e.m. Statistical significance was determined by one-way analysis of variance followed by the Student–Newman–Keuls test. *P* values below 0.05 were considered significant. The percentage of inhibition was calculated by using

100−T×100/C(%) or T×100/C−100(%)

where C and T indicate non-treated (vehicle) and drug-treated, respectively.

## 4. Conclusions

The results obtained in this study confirm the ethnomedicinal use of the ethanol extract from *A. muricata* leaves. The data analysis supported the antinociceptive and anti-inflammatory activities, suggesting a potential for therapeutic purposes. However, further studies should be conducted to ensure its safe usage.

## Figures and Tables

**Table 1.** Effects of the ethanol extract from *A. muricata* leaves on acetic acid-induced writhing in mice.

**Group**	**Dose (mg/kg)**	**Number of writhes**	**Inhibition (%)**
**Control**	Saline	67.62 ± 2.03	-
	100	67.50 ± 1.74	-
**Ethanol Extract**	200	57.87 ± 1.55**	14.42
	400	39.62 ± 1.97***	41.41
**Indomethacin**	10	18.25 ± 0.80***	73.01

Data are mean ± s.e.m. of eight mice.

** *P* < 0.01,

*** *P* < 0.001 *vs.* control group.

**Table 2.** Effects of the ethanol extract from *A. muricata* leaves on formalin-induced nociception in mice.

**Group**	**Dose (mg/kg)**	**Duration of paw licking (s)**			
		**First phase**	**Inhibition (%)**	**Second phase**	**Inhibition (%)**
**Control**	Saline	86.62 ± 3.18	-	93.87 ± 2.73	-
	100	85.87 ± 2.88	-	91.25 ± 3.07	-
**Ethanol Extract**	200	66.12 ± 1.54***	23.67	65.62 ± 1.72***	30.09
	400	47.62 ± 2.13***	45.02	46.75 ± 1.68***	50.20
**Morphine**	1	16.25 ± 1.44***	81.24	19.37 ± 0.94***	79.36

First phase = 0–5 min after formalin injection; second phase = 15–30 min.

Data are mean = s.e.m. of eight mice.

*** *P* < 0.001 *vs.* control group.

**Table 3.** Effects of the ethanol extract from *A. muricata* leaves on the reaction time (s) of mice exposed to the hot-plate test.

**Group**	**Dose (mg/kg)**	**Time after drug administration (s)**			
		**0 min**	**30 min**	**60 min**	**90 min**
Control	Saline	5.50 ± 0.80	6.12 ± 0.44	6.50 ± 0.50	6.75 ± 0.79
	100	5.37 ± 0.80	6.25 ± 0.62	7.12 ± 0.29	7.25 ± 0.45
Ethanol Extract	200	5.50 ± 0.78	6.75 ± 0.45	8.50 ± 0.50*	9.25 ± 0.67*
	400	5.75 ± 0.72	7.37 ± 0.80	11.87 ± 0.64***	13.25 ± 0.84***
Morphine	1	5.75 ± 0.65	9.62 ± 0.82**	13.75 ± 1.10***	16.87 ± 0.93***
Naloxone + Morphine	1 + 1	6.00 ± 0.68	7.87 ± 0.69	8.00 ± 0.46*	7.87 ± 0.55
Naloxone + Extract	1 + 400	5.62 ± 0.68	7.25 ± 0.75	8.75 ± 0,45**	10.87 ± 0.83**

Data are mean ± s.e.m. of eight mice.

* *P* < 0.05,

** *P* < 0.01,

*** *P* < 0.001 *vs.* control group.

**Table 4.** Effects of the ethanol extract from *A. muricata* leaves on carrageenan-induced paw edema in rats.

**Group**	**Dose (mg/kg)**	**Volume of hind paw (mL)**			
		**1 h**	**2 h**	**3 h**	**4 h**
**Control**	Saline	0.53 ± 0.06	0.72 ± 0.05	0.95 ± 0.06	0.75 ± 0.06
	100	0.52 ± 0.09	0.68 ± 0.06	0.80 ± 0.06	0.63 ± 0.04
**Ethanol Extract**	200	0.50 ± 0.10	0.65 ± 0.09	0.73 ± 0.06*	0.53 ± 0.03**
	400	0.48 ± 0.07	0.60 ± 0.04	0.67 ± 0.04**	0.47 ± 0.02***
**Indomethacin**	10	0.47 ± 0.10	0.58 ± 0.05	0.62 ± 0.06**	0.43 ± 0.02***

Data are mean ± s.e.m. of six rats.

* *P* < 0.05,

** *P* < 0.01,

*** *P* < 0.001 *vs.* control group.
